# Evaluation of eMentalHealth.ca, a Canadian Mental Health Website Portal: Mixed Methods Assessment

**DOI:** 10.2196/13639

**Published:** 2019-09-06

**Authors:** Dahn Jeong, Michael Cheng, Mireille St-Jean, Alireza Jalali

**Affiliations:** 1 Faculty of Medicine University of Ottawa Ottawa, ON Canada; 2 Children's Hospital of Eastern Ontario Ottawa, ON Canada; 3 Department of Psychiatry Faculty of Medicine University of Ottawa Ottawa, ON Canada; 4 Department of Family Medicine, Faculty of Medicine University of Ottawa Ottawa, ON Canada; 5 The Ottawa Hospital Ottawa, ON Canada; 6 Division of Clinical and Functional Anatomy Faculty of Medicine University of Ottawa Ottawa, ON Canada

**Keywords:** e-mental health, Canada, online surveys, website assessment

## Abstract

**Background:**

Many Canadians have mental health needs, and it can be challenging not knowing where to go for mental health information, services, and support. The website eMentalHealth.ca was created to facilitate and assist Canadians to (1) learn about mental health, (2) screen for common mental health issues, and (3) find mental health services and support.

**Objective:**

The aim of this study was to use multiple methods to learn about visitors of eMentalHealth.ca, their perceptions, and their satisfaction with the website.

**Methods:**

Website analytics (Google Analytics) provided information about the number of unique visits to the website and how the site was used. Web-based self-administered surveys were used to gather additional information on users’ characteristics and to assess their perception of the website and satisfaction with the website.

**Results:**

Web analytic results showed that from January 1 to December 31, 2017, there were 651,107 users, with 1.97 million page views. Users were more often female than male, and the majority of users were aged 35 years and older. Most users were located in Canada (612,806/651,107, 94.12%), and the most common city of origin of users was Toronto (101,473/651,107, 15.58%), followed by Ottawa (76,692/651,107, 11.78%), and Montreal (26,621/651,107, 4.09%). Web-based surveys were completed by a total of 370 respondents from June to December 2017. Overall, the majority of users were satisfied with the website (93.0%, 320 out of 344 responses). Positive feedback was related to the content of the website as a helpful resource, and negative feedback was related to technical difficulties as well as the design of the main page. This analysis will be used to help the team with ongoing improvements to the website, for example, improving technical issues and homepage usability.

**Conclusions:**

Most visitors reported satisfaction with their use of eMentalHealth.ca to learn about mental health as well as where to find help. Mental health websites such as eMentalHealth.ca are a low-cost way to increase public awareness about mental health.

## Introduction

### Mental Health and E-Mental Health in Canada

In 2012, more than 1 in 6 Canadians reported that they had a need for mental health care in the past 12 months [1]. However, there is a shortage of services and support. The Mental Health Commission of Canada has specifically discussed the importance of *e-mental health* [2] as part of a multipronged strategy to support the mental health needs of Canadians. Electronic mental health (e-mental health) is defined as “mental health services and information delivered or enhanced through the internet and related to technologies” [3].

e-Mental health could transform the current care system in Canada in 2 ways: (1) by empowering patients by providing them with health information and (2) through the use of specific technologies and electronic interventions such as Web-based therapy [2].

A number of studies have conducted an evaluation of informational websites on various mental health topics [4-7]; however, there is a lack of experience with Canadian mental health portals. Hence, it was decided to conduct an evaluation of eMentalHealth.ca using commonly accepted evaluation methods for website evaluations.

### Rationale and Development of eMentalHealth.ca

Launched in 2005 for the general public, eMentalHealth.ca was created to provide reliable information about mental health for Canadians. It is a nonprofit initiative of the Children’s Hospital of Eastern Ontario (CHEO) and the University of Ottawa Brain and Mind Research Institute in Ottawa, Ontario, Canada.

eMentalHealth.ca was created because, before 2005, there was no single Canadian website that provided information about mental health topics, and where to find help.

In 2005, feedback from stakeholders (eg, health care professionals, the general public, and patient and family stakeholders) indicated a lack of credible Web-based information about mental health. The problem included a lack of information on mental health topics and a lack of guidance on when one should be concerned about an individual’s mental health. Stakeholders also noted a lack of *system navigation* tools, that is, difficulty in finding out where to turn for mental health help.

On the basis of this feedback, eMentalHealth.ca was built with the following features:

1. Information about mental health topics and conditions (eg, depression and mental wellness). Articles have been developed by a multidisciplinary team following standard health literacy practices, including stakeholder input (consultation with local organizations such as Parents’ Lifelines of Eastern Ontario, a local parent support group).
2. Web-based screening tools for mental health conditions. Users can fill out validated Web-based screening tools for various mental health conditions anonymously and confidentially. Examples include the Patient Health Questionnaire (PHQ) for adult depression and the Kutcher Adolescent Depression Scale for adolescent depression. Validated screening tools are used when available, that is, tools that have been studied to consistently and reliably predict the presence or absence of a given condition. Tools are used with permission from their rights holders. Tools can be filled out on the Web or printed as PDFs.
3. Where to find mental health help: a searchable directory of mental health providers for major cities and provinces across Canada. The directory includes publicly funded mental health services as well as private services (such as private practice psychologists or psychotherapists). The directory is maintained by a database coordinator who adds new resources as necessary and who also reviews submissions from the general public. Although most mental health websites have information about mental health conditions, not many of them include information about where to get help, which is what many users are seeking [8].

eMentalHealth.ca is bilingual—available in French as approximately 20% of the Canadian population is primarily French speaking [9].

Intellectual property on the website (such as information sheets) is under a Creative Commons license that promotes sharing and collaboration with other mental health agencies. For example, eMentalHealth.ca and Kelty Mental Health Centre in British Columbia collaborated to create a series of Medication Information sheets. Several mental health agencies publish eMentalHealth.ca content on their websites under a Creative Commons license.

To help users see that the site’s information is objective and trustworthy, eMentalHealth.ca is certified as following the Health On Net code (HONcode) Principles from the Health On the Net Foundation. The HONcode certification follows a code of ethics that guides websites to provide quality, objective, and transparent medical information [10].

The original eMentalHealth.ca website was created for the general public. In response to requests from different audiences using the site, 2 additional portals have been created: (1) eMentalHealth.ca/PrimaryCare for primary care providers such as family physicians, nurse practitioners, and pediatricians and (2) eMentalHealth.ca/MedicalStudents for medical students.

### Case Example

Jennifer is a 20-year-old woman who is feeling stressed and tired, and she wonders if she might be having signs of depression. Her family physician is away on holiday, so Jen does what many Canadians do: she uses the internet. She finds the eMentalHealth.ca website and sees that it is a publicly funded hospital and university initiative. She fills out a screening questionnaire for depression (which uses the validated PHQ-9 scale) [11], and it shows that she may have depression. The website recommends speaking to a health care provider and provides more information and resources about depression, including local mental health services. She has not yet seen her family physician, but she is feeling more confident about what steps to take to cope with her situation.

### Rationale and Novel Aspects of This Evaluation

Following the launch of eMentalHealth.ca, there have been regular updates to the website, based on informal feedback from users and results from internal usability testing sessions. When launched, eMentalHealth.ca was unique. Over time, there has been an explosion of mental health websites, making it hard for the average user to evaluate which websites are reliable. It was thus decided to perform a formal, published evaluation to help the general public ensure that the website was credible. Key questions for the evaluation included: Who is visiting the site? What are their experiences, positive and negative? Why are they visiting the site? From where are they visiting the site? What are the ways we can improve?

## Methods

### Web Analytics

Google Analytics was used to determine the number of users and the number of sessions since the launch of each website as well as demographic information about the visitors, including the countries and cities associated with the users, the frequency and recency of the sessions, and the top site contents viewed. Google Analytics is a widely used Web analytics service offered by Google that tracks and reports website traffic [12].

### Self-Administered Surveys

Web-based self-administered surveys were developed to gather data on users’ characteristics and other standard questions used in website evaluations. Questionnaires were based on the Commission of the European Communities’ quality criteria for health-related websites [13] and on studies by Tlach et al [4] and Kuosmanen et al [5]. The surveys were reviewed and piloted internally by hospital and university colleagues who did not have background information on the website evaluation project. A weekly draw for a Can $50 gift certificate from Amazon.ca was held for 8 weeks to encourage survey participation. The following items of the Checklist for Reporting Results of Internet E-Surveys Checklist [14] for reporting electronic survey results were also considered: design, Institutional Review Board approval and informed consent process, development and pretesting, recruitment process and description of the sample having access to the questionnaire, and survey administration and analysis.

To view a copy of the questionnaire, one can visit this link: https://www.surveymonkey.com/r/T8GSQBN. The surveys collected general demographic information such as gender, age, and type of user (general public, student or health professional, eg, family physician, nurse practitioner, psychiatrist, or other). In addition, questions assessed satisfaction with the design and content of the website, frequency of use, users’ perceived trust about the information, and future intentions. All questions were Likert-type scale responses. At the end of the survey, there was 1 open-ended question for any comments about the websites, and respondents were also asked if they would like to be contacted to participate in a focus group. The responses to the open-ended question were single coded by 1 coder. The open-ended comments were used to provide overall and general insight into the users’ experience. In the scope of this study, this analysis was not aimed at deeply understanding the qualitative experience of the users. Emerging themes and comments were assessed.

For the English version of the website, 53 respondents provided a viable comment (out of 207 respondents), and for the French version, 17 respondents provided a viable comment (out of 153 respondents).

The collected data on user characteristics and users’ satisfaction were analyzed on SAS statistical software [15] using descriptive statistics, and the responses to the 1 open-ended question were analyzed by qualitative content analysis [16] using NVivo qualitative analysis software [17].

### Ethics

An ethics application was submitted to and reviewed by the Ottawa Health Science Network Research Ethics Board. The review indicated that the project fell within the quality initiative, quality improvement, quality assurance, and/or program evaluation category, and a confirmation letter was received.

## Results

Web-based surveys were posted on the main page of eMentalHealth.ca and eSantéMentale.ca, and the survey results included in this paper were collected from June to November 2017.

### Google Analytics

Table 1 summarizes the Web analytics (from Google Analytics). There were more female users than male users, and the majority of users were 35 years and older. These two demographic characteristics are also reflected in the survey results. The majority of visitors to the websites were new visitors, accounting for 87.11% (643,100/738,291) to 89.16% (221,857/248,825) of the visitors and were from Canada (93.4% for English website and 54.4% for French website). For the French version, over one-third of the visits were from France. Within Canada, most users were from Ontario, reflecting the project’s home base in Ontario. As for the preferred content, *Mental Health Screening Tools* (*Outils de dépistage* in French) was one of the top 5 viewed content. However, on the English site, resources were the most viewed items, whereas on the French site, screening tools were the most viewed items. Finally, regarding the devices used to access the website, the majority of users of eMentalHealth.ca used desktop computers (54.40%, 353,375/649,541), followed by mobile devices (37.60%, 244,248/649,541) and tablets (7.99%, 51,918/649,541). Users of the French version were accessing the website with mobile devices and desktop equally often (44.44%, 99,945/224,879). (Note that the website is mobile friendly with a liquid layout.)

**Table 1:** Google Analytics results for eMentalHealth.ca and eSantéMentale.ca

Analytics		eMentalHealth.ca	eSantéMentale.ca
Timeframe		January 1-December 31, 2017	January 1-December 31, 2017
Users/year		651,107	222,757
Users/month		54,000	18,500
Page views		1,968,778	469,933
**Gender, n (%)**			
	Female	286,792 (72.88)	52,500 (70.02)
	Male	106,695 (27.12)	22,478 (29.98)
**Age (years), n (%)**			
	18-24	63,356 (16.01)	13,666 (18.91)
	25-34	115,371 (29.15)	20,052 (27.75)
	35-44	87,659 (22.15)	17,744 (24.56)
	45-54	70,736 (17.87)	10,471 (14.49)
	55-64	40,466 (10.22)	6,531 (9.04)
	65+	18,241 (4.61)	3,788 (5.24)
**Top countries, n (%)**			
	Canada	612,806 (94.12)	121,332 (54.47)
	United States	21,538 (3.31)	—^a^
	United Kingdom	4,703 (0.72)	—
	France	—	70,720 (31.75)
	Belgium	—	10,221 (4.59)
**Top cities, n (%)**			
	Toronto	101,473 (15.58)	—
	Ottawa	76,692 (11.78)	—
	Montreal	26,621 (4.09)	32,936 (14.79)
	Paris	—	17,983 (8.07)
	Quebec City	—	10,669 (4.79)
New visitors, n (%)		643,100 (87.11)	221,857 (89.16)
Returning visitors, n (%)		95,191 (12.89)	26,968 (10.84)
Top site content viewed		Mental Health Resources, Help and Support In Your Community	Outil de dépistage: Schizophrénie
		Search eMentalHealth.ca	Outil de dépistage: Dépression
		Group Homes, Residential Care and Supported Housing: Ontario	Outils de dépistage
		Mental Health Screening Tools	Buproprion (Wellbutrin)
		Mental Health Events Calendar	Ressources, aide et support en santé mentale dans votre communauté
**Device of access to website, n (%)**			
	Desktop	353,375 (54.40)	98,895 (43.98)
	Mobile	244,248 (37.60)	99,945 (44.44)
	Tablet	51,918 (7.99)	26,039 (11.58)

^a^Empty cells are filled with a dash for visual clarity.

### User Survey Results

Table 2 presents the results of the user surveys for eMentalHealth.ca and eSantéMentale.ca. Consistent with the Google Analytics results, there were more users that identified themselves as female than male, and the majority of users were 31 years and older. Most survey respondents (68.5%, 141/207 and 77.78%, 119/153 for eMentalHealth.ca and eSantéMentale.ca, respectively) were from the general public, matching the surveys’ intended target audience. Significant numbers identified themselves as health care professionals (20.29%, 42/207 in eMentalHealth.ca and 16.9%, 26/153 in eSantéMentale.ca). There were also significant numbers of health care professionals included in this survey: 20.29% in eMentalHealth.ca and 16.99% in eSantéMentale.ca. Regarding the frequency of use, for eMentalHealth.ca, the majority (56.1%, 115/207) of respondents indicated that they have used the website from 2 to 4 times. For eSantéMentale.ca, the majority of respondents were first-time users of the website. This variation might be related to how long each website has been on the Web, as the eMentalHealth.ca has been active since 2005, whereas the eSantéMentale.ca launched later in 2010.

**Table 2:** Descriptive characteristics and frequency distribution of users for eMentalHealth.ca (N=207) and eSantéMentale.ca (N=153).

Characteristic		eMentalHealth.ca, n (%)	eSantéMentale.ca, n (%)
**Gender**			
	Female	163 (78.7)	82 (54.0)
	Male	40 (19.3)	70 (46.0)
	Prefer not to say	4 (2.0)	0
**Age (years)**			
	Under 18	1 (0.5)	3 (2.0)
	18-30	39 (18.8)	31 (20.4)
	31-40	50 (24.2)	37 (24.3)
	41-50	59 (28.5)	38 (25.0)
	51-60	44 (21.3)	28 (18.4)
	Over 60	14 (6.8)	15 (9.9)
**Occupation**			
	Health care professionals	42 (20.3)	26 (17.0)
	Students and research professionals	6 (2.9)	2 (1.3)
	Social workers and counsellors	17 (8.2)	6 (3.9)
	General public	141 (68.5)	119 (77.8)
**Frequency of use**			
	First time	20 (9.8)	121 (79.6)
	Less than 5 times	115 (56.1)	15 (9.9)
	Between 5 and 9 times	26 (12.7)	8 (5.3)
	More than 10 times	44 (21.5)	8 (5.3)

### Users’ Satisfaction With the Websites

**Figure 1:**
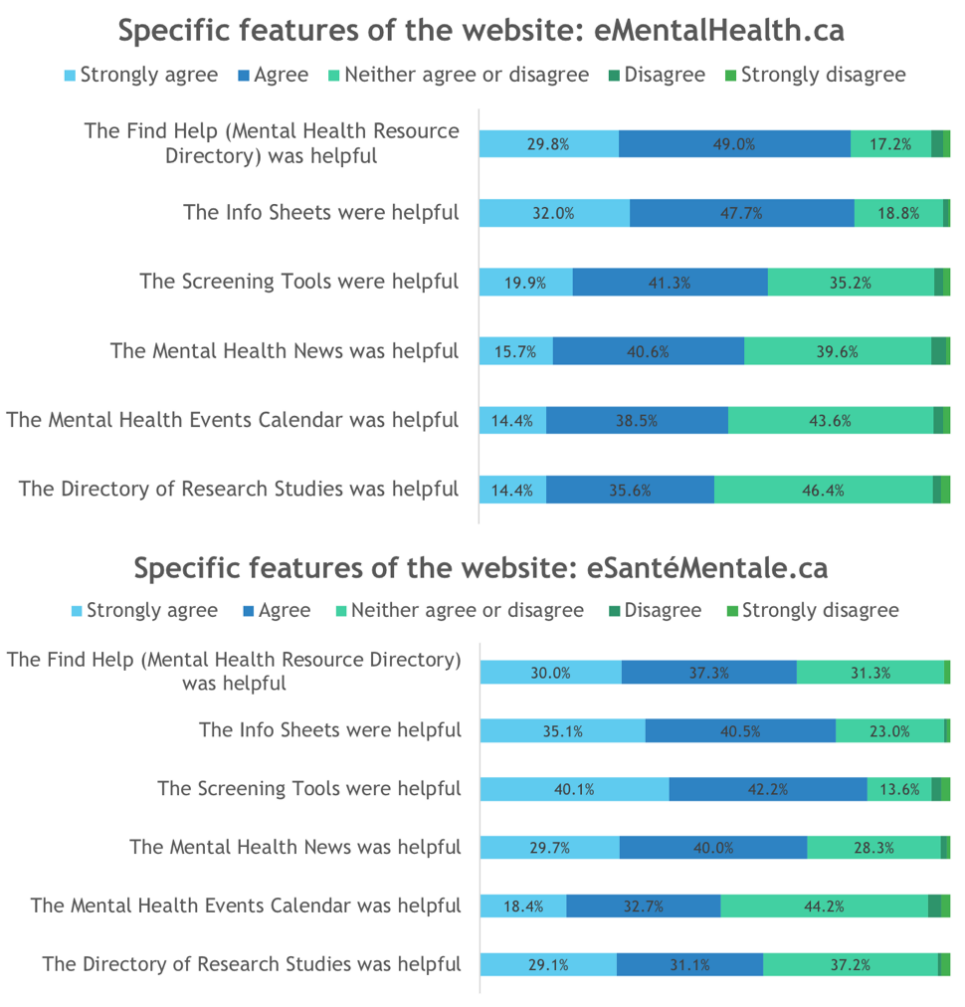
Users’ ratings of specific features of the websites eMentalHealth.ca (n=207) and eSantéMentale.ca (n=153).

In the surveys, users were asked to rate the specific features of the websites (eMentalHealth.ca and eSantéMentale.ca). They were also asked to rate statements to measure the users’ satisfaction with website design and content, perceived trust, frequency of use, and future intentions to use the website. Figure 1 demonstrates users’ ratings of specific features of the websites.

**Figure 2:**
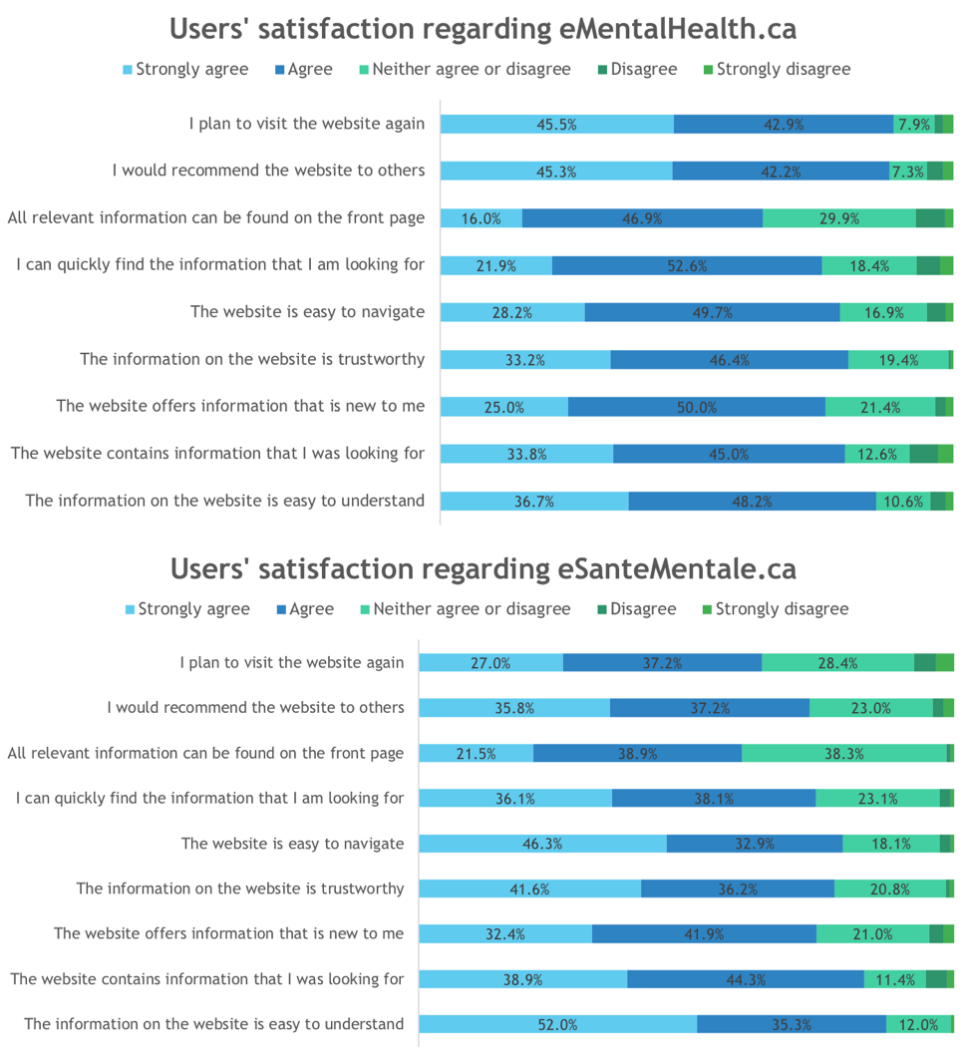
Users’ satisfaction with the websites eMentalHealth.ca (n=207) and eSantéMentale.ca (n=153).

Figure 2 presents users’ satisfaction with website design and content, perceived trust, and future intentions to use the websites.

### Were the Website Features Helpful?

Most users of eMentalHealth.ca and eSantéMentale.ca found the specific features of the websites helpful: close to 80% of users strongly agreed or agreed that the Find Help and Info Sheets were helpful to them; and over 50% of users strongly agreed or agreed that the rest of the features (Screening Tools, Mental Health News, Mental Health Events Calendar, and Directory of Research Studies) were helpful to them. In all the surveys, very few users (less than 5%) disagreed or strongly disagreed that the specific features of the websites were helpful to them.

### Were the Users Satisfied With the Website?

Most users of eMentalHealth.ca and eSantéMentale.ca reported being satisfied with the websites. For eMentalHealth.ca, more than 75% of users strongly agreed or agreed with each statement except one: just 63% of users strongly agreed or agreed that “All relevant information can be found on the front page.” In addition, 88% of users would recommend the website to others and were planning to visit the website again. Similarly, for eSantéMentale.ca, more than 70% of users strongly agreed or agreed to each statement except 2 statements: 60% of users strongly agreed or agreed that “All relevant information can be found on the front page” (“Toutes les informations pertinentes se trouvent sur la page principale”), and 64% strongly agreed or agreed that “I plan to visit the website again” (“Je planifie de visiter le site web sous peu”).

### Overall Satisfaction With the Websites

In both surveys, 90% or more of respondents were very satisfied or somewhat satisfied with the website overall (eMentalHealth.ca and eSantéMentale.ca). Table 3 presents the overall satisfaction rating by the users of the websites.

#### Open-Ended Comments From Users

Survey respondents left comments at the end of the survey. Most comments were positive, stating that the website was a good resource for finding helpful mental health information and services. A few examples are shown in Table 4.

Negative comments were very helpful most of the time as they had clear suggestions on how the website could be improved. Mainly, comments were related to difficulties with setting the location of interest. Respondents also mentioned that the main page could be too busy and that some information on the website was outdated. Several examples of comments are shown in Table 5.

**Table 3:** Overall satisfaction with the websites eMentalHealth.ca (N=199) and eSanteMentale.ca (N=145).

Website	Very satisfied, n (%)	Somewhat satisfied, n (%)	Somewhat dissatisfied, n (%)	Very dissatisfied, n (%)
eMentalHealth.ca	119 (59.8)	60 (30.2)	13 (6.5)	7 (3.5)
eSanteMentale.ca	46 (31.7)	95 (64.8)	4 (2.8)	1 (0.7)

**Table 4:** Open-ended comments from users—positives.

Comment	User type
“This is a great initiative because generally it is hard to find up to date information for resources in some areas...”	Researcher
“To help bridge the gap between assessment and treatment, I print off the info sheets to provide to parents during debrief.”	Clinical psychologist
“This website was surprisingly well-equipped to provide well-rounded groupings of information on mental health.”	Caretaker (general public)
“Overall, this is an excellent resource for clients and other professionals who are researching where to find specific services.”	Psychologist
“This is a fabulous resource. Thank you!”	Social Worker: psychotherapy
“The directory is a very good and helpful resource.”	General public
“Very informative. Glad this tool is available for the community. It would be nice to see an info sheet and/or screening tool for compassion fatigue.”	Counsellor/psychometrist
“This is the most comprehensive website for mental health resources. I use it regularly and recommend it to staff and patients.”	Counsellor

**Table 5:** Open-ended comments from users—negatives.

Comment		User type
**Improving the design, especially of the homepage**		
	“The design is not very nice. The style itself is a little old.” (translated from French) and “A lot of information at once (visually) on the main page.” (translated from French)	General public
	“Perhaps an even simpler design for the site, there is a lot of information presented at once. For the general public the main purpose is showing them resources, so everything to the right with the news and tips doesn't need to be in the locations where they are. The main buttons under ‘find mental health help in your area’ is all you need there, because sometimes maybe things are repeated and someone may not know which one to click.”	Researcher
	“A little confusing at first as there is a lot of information on the front page...”	General public
	“The website front page could use a serious design overhaul. It is too wordy...overall too much going on making it difficult to navigate.”	Psychotherapist
**Info sheets**		
	“The info sheet for depression was simplistic.”	General public
	“Info sheets are best part of the website but they are in general too long (try for 2 pages max).”	Psychiatrist
**Improving the resource directory**		
	“Little info about my hometown (Pembroke) but that may be because there is little info about counselling/help offered here. An alternative could be online counselling or resources via skype, IM offered by hospitals for this isolated and hopeless rural hell and other similar hells where people are isolated and can’t get out of. There is no public transit here so accessing resources even across town is difficult for many.”	General public
	“Many of the links offered are in cities other than Calgary; in fact, many of them are in Toronto.” (User from Calgary.)	Psychologist
**Improving findability of local resources**		
	“Location based search doesn’t narrow down location as it should and mental health directory has too many search results and are too broad.”	Psychiatrist

## Discussion

### Principal Findings

The goals of this study were to collect information about the websites’ users and to describe the user population, as well as to assess the users’ satisfaction with the websites in terms of design and content, perceived trust, frequency of use, and future intentions to use the website.

First, as predicted by the Google Analytics results, the user population was, in majority, female and aged 31 years and older. Most users of the English and French websites were categorized as members of the general public, which is the targeted user population of the websites. About 90% of users of the English website had visited the website more than once. The French website had more first-time users. Google Analytics is a simple, free way to gather demographic information about users, whereas Web surveys are much more labor intensive. As shown in our surveys, the demographic results of Google Analytics were representative of what Web surveys reported. For future surveys, one could use Google Analytics for demographic data such as gender, age, language, and location of users, and the more burdensome Web survey could be shortened.

In terms of user satisfaction by language, it was noted that more English users were *very satisfied* than French users (59.8% of English users vs 31.7% of French users). Conversely, it was easier to make French users *somewhat satisfied* at 64.8% versus English users at 30.2%. It was unclear why this might be. However, the total numbers that were *very satisfied* and *satisfied* were rather similar—English users at 90.0% and French users at 96.5%.

Website survey results showed that the most highly related features were (a) the Mental Health Resource Directory listing mental health services and (b) Info Sheets about mental health conditions and topics. These are the areas that will be prioritized in the ongoing improvement of the website.

The open-ended comments from surveys showed that most respondents felt positively about the website. There were consistent themes, such as appreciating the resources directory, the information sheets, and screening tools. The project team was concerned whether entering users in a draw for a prize might create mainly positive feedback, but fortunately, this was not the case. There was a good amount of constructive negative feedback from the survey. Themes included complaints about the home page usability, findability, and searchability of the website. Negative feedback is most valuable for making changes and improvements to the website.

Top areas for improvement might include the following:

1. Improving the home page to make it less overwhelming and easier to use. The literature on home page usability recommends reducing the number of clicks [18]. Given that info sheets are popular, it might be helpful to make top info sheets clickable from the home page.
2. Improving the resource directory. Key resources should be included for major cities in Canada.
3. Further exploration of how to improve the mental health info sheets. Some users wanted briefer versions, whereas other users wanted more detailed information. Different users may have different information needs, and it might be helpful to have both brief and longer versions of info sheets.

This evaluation will help with improving the English and French websites for their users, in the ongoing effort to provide accessible, trustworthy, and credible information for those with mental health needs and for the family members and professionals who support them.

### Limitations

There were a number of limitations to this study. First, small sample size of the respondents to the Web-based self-administered surveys caused by a low participation rate means the survey results may not be representative of all website users. For example, many users suggested that the home page should be improved. Hopefully, they are representative of most users and not simply a vocal few. One way to address this in a future study (after the website is updated) would be having a long Web survey period. Second, challenges remain in distinguishing expectations and preferences for the different user populations. eMentalHealth.ca targets a wide audience, including the general public, primary care providers, and medical students. Web survey results were positive for all groups of users, and there were many similarities in the types of feedback, whether users were from the general public or professionals. It may be that larger numbers of surveys would be required to see the differences between the different user groups or perhaps a different form of data collection such as focus groups or individual interviews. Third, the time frames of data collection for website surveys and Google analytics data were not same. Google analytics data were collected for January 1 to December 31, 2017, and it would have been ideal to collect Web surveys over the same time period. Unfortunately, this was not possible because of a lack of resources to gather Web surveys over that time period (lack of funds for a weekly draw for a whole year, and lack of funds for research coordinator time). For future evaluations, efforts will be made to secure more resources for a longer period of Web-based surveys. Finally, in an effort to make the Web survey questionnaire as brief as possible, there were few open-ended fields. In the end, however, the open-ended fields were extremely valuable, including negative feedback. In the next evaluations, it would be greatly helpful for continuous quality improvement of the websites to have more open-ended fields and/or to provide the option of calling survey participants for a follow-up interview to solicit more detailed comments.

### Conclusions

eMentalHealth.ca and eSantéMentale.ca were viewed positively by most users. Users appreciated the mental health information, screening tools, and *where to find mental health* resources. However, users expressed concerns about technical issues, including homepage usability (ie, finding the homepage cluttered). Feedback from this evaluation will be used to upgrade and improve future versions of the website, which will help both the general public and the professionals that use the website.

## Abbreviations

CHEO: Children’s Hospital of Eastern Ontario
e-mental health: electronic mental health
HONcode: Health On Net code
PHQ: Patient Health Questionnaire

## References

[1] Sunderland A, Findlay LC (2013). Perceived need for mental health care in Canada: results from the 2012 Canadian community health survey-mental health. Health Rep.

[2] Mental Health Commission of Canada.

[3] Christensen H, Griffiths KM, Evans K (2002). Centre for Mental Health Research The Australian National University.

[4] Tlach L, Thiel J, Härter M, Liebherz S, Dirmaier J (2016). Acceptance of the German e-mental health portal www.psychenet.de: an online survey. PeerJ.

[5] Kuosmanen L, Jakobsson T, Hyttinen J, Koivunen M, Välimäki M (2010). Usability evaluation of a web-based patient information system for individuals with severe mental health problems. J Adv Nurs.

[6] Young M, Richards C, Gunning M (2012). Online mental health resources for teenagers: an evaluation of two websites developed for adolescents. Adv Sch Ment Health Promot.

[7] Berk L, Berk M, Dodd S, Kelly C, Cvetkovski S, Jorm AF (2013). Evaluation of the acceptability and usefulness of an information website for caregivers of people with bipolar disorder. BMC Med.

[8] Wetterlin FM, Mar MY, Neilson EK, Werker GR, Krausz M (2014). eMental health experiences and expectations: a survey of youths' web-based resource preferences in Canada. J Med Internet Res.

[9] (2017). Statistics Canada.

[10] (1997). Health On the Net Foundation.

[11] Arroll B, Goodyear-Smith F, Crengle S, Gunn J, Kerse N, Fishman T, Falloon K, Hatcher S (2010). Validation of PHQ-2 and PHQ-9 to screen for major depression in the primary care population. Ann Fam Med.

[12] Clark D, Nicholas D, Jamali Hr (2014). Evaluating information seeking and use in the changing virtual world: the emerging role of Google Analytics. Learn Pub.

[13] Commission of the European Communities‚ Brussels (2002). eEurope 2002: quality criteria for health related websites. J Med Internet Res.

[14] Eysenbach G (2004). Improving the quality of web surveys: the checklist for reporting results of internet e-surveys (CHERRIES). J Med Internet Res.

[15] (2017). SAS.

[16] Hsieh HF, Shannon SE (2005). Three approaches to qualitative content analysis. Qual Health Res.

[17] QSR International.

[18] Nielsen J (2002). Nielsen Norman Group.

